# Irritable Bowel Syndrome in Inflammatory Bowel Disease: An Evidence-Based Practical Review

**DOI:** 10.3390/jcm15010116

**Published:** 2025-12-24

**Authors:** Mohsin F. Butt, Mustafa H. Reghefaoui, Aaron Shailesh Benedict, Maiss Reghefaoui, Hussain Al-Jabir, Aneeqa Shaikh, Katarina Vojtekova, Gordon W. Moran, Maura Corsetti, Qasim Aziz

**Affiliations:** 1 NIHR Nottingham Biomedical Research Centre, Nottingham University Hospitals NHS Trust and The University of Nottingham, Nottingham NG7 2RD, UK; 2 Wingate Institute of Neurogastroenterology, Centre for Neuroscience, Trauma and Surgery, Blizard Institute, Barts and The London School of Medicine and Dentistry, Queen Mary University of London, London E1 2AJ, UK; 3 Department of Internal Medicine, University of Debrecen, 4032 Debrecen, Hungary; 4 Department for Pain Medicine, East Kent Hospitals University NHS Foundation Trust, Kent CT1 3NG, UK

**Keywords:** abdominal pain, irritable bowel syndrome, inflammatory bowel disease

## Abstract

Irritable bowel syndrome (IBS) is a disorder of gut–brain interaction characterized by recurrent abdominal pain associated with a change in the frequency and/or form of stools. Approximately one in three patients with quiescent inflammatory bowel disease (IBD), defined as the absence of endoscopic evidence of active inflammation, experience IBS-type symptoms. These symptoms are associated with reduced quality of life and increased psychological burden, and can complicate clinical assessment by mimicking conditions such as small intestinal bacterial overgrowth, bile acid malabsorption, or post-inflammatory complications. This up-to-date narrative review examines the mechanisms, diagnostic challenges, and management of IBS-type symptoms in quiescent IBD. Evidence suggests that these symptoms arise from a complex “matrimony” of functional and organic processes, including low-grade residual inflammation, altered intestinal permeability, microbiota dysbiosis, visceral hypersensitivity, and psychosocial impairment. Diagnosing IBS-type symptoms in IBD requires a “positive”, symptom-focused approach while carefully excluding active inflammation. Management should adopt a biopsychosocial approach, integrating dietary strategies (e.g., low-FODMAP diet), brain–gut behavioral therapy, biofeedback therapy, and/or pharmacological treatments such as antispasmodics, antidiarrheals, laxatives, and neuromodulators to address both physiological and psychological factors. Future research should integrate sensitive biomarkers and longitudinal follow-up to enhance diagnostic precision and guide personalized therapy. Understanding and addressing the overlap between IBS and IBD is essential to reduce the multidimensional burden on physical health, psychological well-being, and daily functioning.

## 1. Introduction

**Figure 1:**
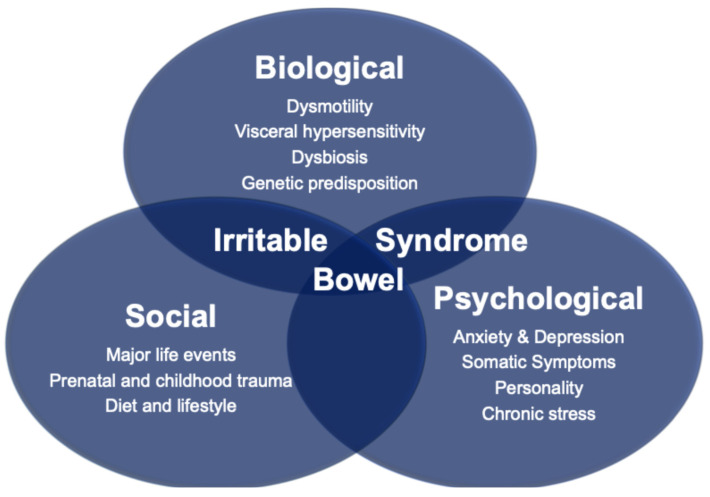
The Biopsychosocial Model of Irritable Bowel Syndrome.

Rome IV IBS, which affects, respectively, 4.3% of the UK and 4.1% of the global population [1,2], is a disorder of gut–brain interaction (DGBI) characterized by altered bowel movements (either constipation, diarrhea, or both) and abdominal pain that is either relieved or worsened with defecation [3]. DGBIs refer to chronic abdominal symptoms which do not have an identifiable structural or biochemical cause using routine medical investigations. As with other DGBIs, the etiopathogenesis of IBS can be conceptualized through the biopsychosocial model, which addresses the dynamic interplay between biological, psychological and social factors in the genesis and evolution of the disease (Figure 1).

Inflammatory bowel disease (IBD) is a chronic inflammatory disease of the gastrointestinal tract, which can be broadly divided into two main subtypes: Crohn’s disease and ulcerative colitis. These two conditions differ micro- and macroscopically and vary with respect to the location of inflammation, depth of involvement of the bowel wall, and symptom profile [4]. Much like IBS, the etiology of IBD remains incompletely understood, with genetic susceptibility and dysregulated immune responses among the leading proposed mechanisms. IBD has an estimated worldwide population prevalence of 0.3% [5] and is predicted to be as high as 1% by 2030 [6].

The overall aim of this review is to provide an overview of the etiopathogenesis and management of IBS in IBD. The authors do not intend to provide an exhaustive overview of IBS in IBD, as this has been addressed elsewhere [7,8,9,10], but instead highlight salient issues for the general physician.

## 2. Challenging Transitional Perspectives

According to traditional dogma, IBS should only be diagnosed in patients who have no organic gastrointestinal pathology, such as IBD, that could explain symptoms. A more nuanced perspective, and one that is gaining increasing traction within mainstream neurogastroenterology practice, is that symptoms of IBS may disproportionately manifest relative to the observed disease activity in IBD. In other words, the degree of observable mucosal inflammation is insufficient to explain gastrointestinal symptoms. This blurring of boundaries between “functional” and “organic” pathologies was described as early as 1983 by Whorwell et al. [11] who reported that 33% of patients with ulcerative colitis in remission (defined as being off all medications except maintenance sulfasalazine and the absence of blood, mucus or contact bleeding on a flexible sigmoidoscope) met the symptom-based criteria for IBS.

## 3. Obstacles to Understanding IBS in IBD

### 3.1. Distinguishing Active IBD from IBS in IBD

**Figure 2:**
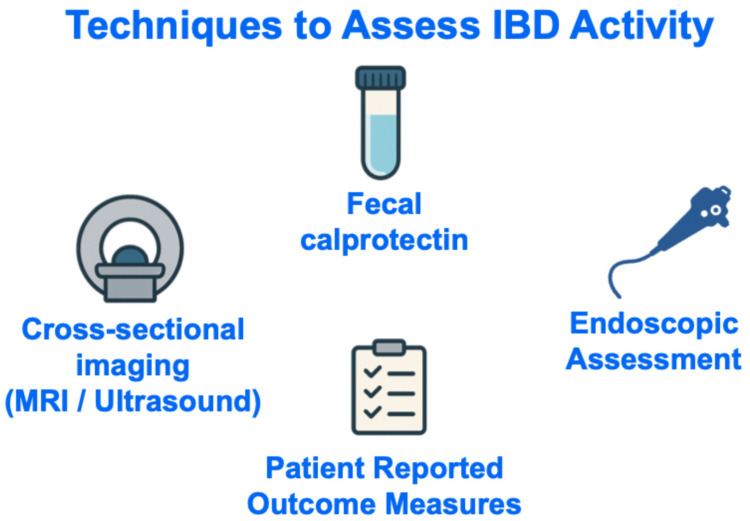
Techniques to Assess IBD Activity. Abbreviations: IBD, inflammatory bowel disease; MRI, magnetic resonance imaging.

Numerous strategies may be used to track disease activity in IBD [12,13], but four that are perhaps most ubiquitously used in clinical practice worldwide are measurement of fecal calprotectin, cross-sectional imaging, endoscopic visualization of the gastrointestinal mucosa, and/or assessment of patient-reported outcomes (Figure 2).

#### 3.1.1. Fecal Calprotectin

Fecal calprotectin is a marker of neutrophil migration into gastrointestinal tissue [14], and is endorsed by the British Society of Gastroenterology as a validated, non-invasive biomarker for endoscopic and histological inflammatory activity in IBD [13,15]. The sensitivity and specificity of fecal calprotectin in identifying active disease can vary according to proposed cut-off value. One meta-analysis ascribed optimum sensitivity (90.6%) to levels > 50 μg/g, whereas optimum specificity (78.2%) to levels > 100 μg/g [16]. The American Gastroenterological Society uses a fecal calprotectin cut-off of 150 μg/g to determine whether gastrointestinal symptoms are related to an exacerbation of inflammation in Crohn’s disease [17] or ulcerative colitis [18]. Importantly, fecal calprotectin may not be sensitive to small bowel inflammation, which may feature in Crohn’s disease, hence a normal level does not exclude mucosal inflammation [19,20].

#### 3.1.2. Cross-Sectional Imaging

Cross-sectional imaging techniques, such as magnetic resonance enterography and intestinal ultrasound, allow complete visualization of the small bowel and assessment for extraintestinal disease in IBD [21]. Magnetic resonance imaging is considered the gold-standard imaging technique for monitoring small bowel Crohn’s disease [22]. Over the past two decades, increasing attention has been paid to measuring small intestinal motility using this modality [23], which is helpful for the assessment of inflammatory burden [24], but is not predictive of response or remission to biological therapy at one year [25].

Considering the extended wait times and significant expenses linked to serial magnetic resonance imaging, bedside intestinal ultrasound has gained prominence as a cost-efficient, rapid, and better-tolerated tool for monitoring individuals with IBD [26]. A recent systematic review identified 23 intestinal ultrasound scores developed for both Crohn’s disease and ulcerative colitis [27], with endoscopy serving as the gold standard in 83% of the studies. Of these scores, 13 had undergone validation, but only 7–8 were validated in more than two independent cohorts. The parameters most frequently incorporated into the scoring systems included bowel wall thickness, vascularity, bowel wall stratification, and inflammatory fat [27].

Bedside intestinal ultrasound serves as a crucial outcome tool in IBD, both informing treatment response, providing prognostic insights, and guiding clinical decision-making. For instance, early intestinal ultrasound response has been shown to predict long-term outcomes to biological therapy in IBD; ultrasound remission (Milan ultrasound criteria score < 6.2) in ulcerative colitis at week 12 following biological treatment independently predicts long-term endoscopic improvement or remission [28], while lack of ultrasound improvement after one year of anti-tumor necrosis factor therapy in Crohn’s disease is associated with higher rates of surgery and therapy escalation [29]. Bedside intestinal ultrasound also directly influences outcomes through improved clinical decision-making, as demonstrated by an observational study in which its use led to acute treatment changes in 57% of patients with Crohn’s disease and avoided urgent endoscopy in 85% of cases [30].

#### 3.1.3. Endoscopic Assessment

The Mayo Disease Activity Index for ulcerative colitis [31] is widely employed in contemporary clinical practice [15,31,32], with its endoscopic subscore derived from assessment of mucosal erythema, vascular pattern and friability. The original description of the Mayo Score also included two patient-reported outcomes (stool frequency and rectal bleeding) and a physician’s global assessment [31]. Other working groups have suggested the Ulcerative Colitis Endoscopic Index of Severity [33], the Crohn’s Disease Endoscopic Index of Severity [34], and the Simple Endoscopic Score for Crohn’s Disease [35] as alternative scoring tools.

#### 3.1.4. Histological Assessment

The lack of an agreed definition of histological remission in ulcerative colitis and Crohn’s disease renders this an adjunctive, rather than primary, goal in the management of IBD [32]. The prognostic benefits of histological remission have yet to be confirmed [36].

#### 3.1.5. Patient-Reported Outcome Measures (PROMs)

Twenty different PROMs have been proposed to measure disease activity in IBD, among which none meet the Food and Drug Administration (FDA) recommended criteria, and only two—the Harvey–Bradshaw Index and Simple Clinical Colitis Activity Index scores—have been developed with patient input [37]. PROMs may be influenced by depression and anxiety, and patients with more severe depressive symptoms are more likely to be classified as having active IBD [38,39], highlighting the need to interpret PROM-determined disease activity in the context of psychological symptoms. The recently developed PROMS, namely the PRO2-Ulcerative Colitis (which assesses stool frequency and rectal bleeding) and the PRO2-Crohn’s Disease (which assesses stool frequency and abdominal pain), are likely to be less influenced by comorbid psychopathology [40].

### 3.2. Determining the Prevalence of IBS in IBD

A proportion of patients labelled as having quiescent IBD with IBS-type symptoms may have subclinical or low-grade inflammatory activity that escapes detection by standard tools, leaving the rate of “true” IBS in IBD uncertain. Endoscopic evaluation is considered the “gold standard” method for assessing mucosal inflammation [9] and the prevalence of IBS-type symptoms is significantly lower using this modality (23.5%) compared with disease activity indices or fecal calprotectin (33.6% and 35.1%, respectively) [41]. Despite this, endoscopy and fecal calprotectin both remain imperfect surrogates for mucosal immune activation. Indeed, microscopic inflammation, altered permeability, or cytokine activity may persist even when tests suggest remission [42,43]. Overall, the pooled prevalence of IBS symptoms in IBD is estimated to be 32.5% (95% CI 27.4–37.9), with a higher prevalence among patients with Crohn’s disease versus ulcerative colitis [41]. In a multivariate Cox regression model, Crohn’s disease involvement outside the ileum or colon conferred the highest increased risk of IBS-type symptoms in quiescent CD (HR 20.1 [95% CI 2.5–160.7], *p* = 0.005) [44].

**Figure 3:**
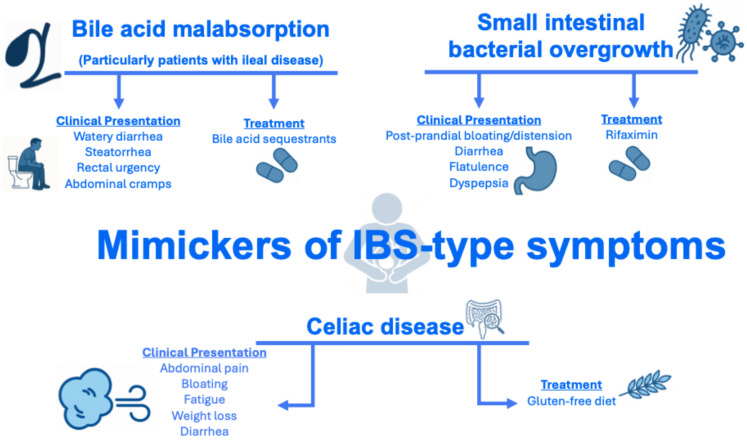
Conditions that may mimic IBS in patients with IBD. Abbreviations: IBD, inflammatory bowel disease; IBS, irritable bowel syndrome; SIBO, small intestinal bacterial overgrowth.

The prevalence of IBS-type symptoms in IBD is also dependent on the diagnostic tool used to assess IBS. IBS can be diagnosed using symptom-derived frameworks, such as the Manning and Rome (I–IV) criteria [45], which were originally developed for use in individuals without organic gastrointestinal disease. Their application to patients with IBD can potentially be problematic. Indeed, among patients with IBD, symptoms that meet Rome criteria for IBS may in fact reflect alternative pathophysiological processes that patients with IBD are predisposed to, rather than a true DGBI. For instance, small bowel involvement in Crohn’s disease may increase susceptibility to small intestinal bacterial overgrowth (SIBO) [46], whereas terminal ileal disease or resection may lead to bile acid malabsorption [47] (Figure 3).

## 4. The Etiology of IBS in IBD

DGBIs, such as IBS, arise secondary to a combination of altered motility, visceral hypersensitivity, epithelial barrier disruption, mucosal immune dysfunction, microbiota alteration, or gut–central nervous system neural processing [48]. IBD has been shown to affect each of these elements, both in active and quiescent disease states, and such shared mechanisms may contribute to the association between IBS and IBD.

### 4.1. Altered Motility and Visceral Hypersensitivity

Two key studies have investigated intestinal motility in IBD [49,50]. Compared with healthy controls, patients with ulcerative colitis exhibit decreased colonic contractility, increased low-amplitude propagating colonic contractions, and variable colonic transit [49]. Resting rectal motor activity is demonstrably diminished among patients with ulcerative colitis versus healthy controls; however, luminal distension can cause the inflamed rectum to generate abnormally strong contractions that may threaten continence [50].

Active colitis has been associated with significantly reduced rectal compliance, that is, a reduction in the ability of the rectum to stretch and expand to accommodate stool, but not in patients with quiescent colitis compared with healthy controls [51]. This suggests that frequent and urgent defecation in patients with active ulcerative colitis may be related to a poorly compliant and hypersensitive rectum.

### 4.2. Epithelial Barrier Disruption and Mucosal Immune Dysfunction

A lower expression of markers of epithelial barrier integrity (that is, ZO-1 and α-catenin) taken from tissue biopsies has been reported among patients with both IBS and quiescent IBD with IBS-type symptoms compared with patients with quiescent IBD without IBS-type symptoms or healthy controls [42]. This is consistent with the finding of increased colonic paracellular permeability in both patients with quiescent ulcerative colitis and, to a lesser degree, in IBS-mixed-subtype patients [43]. Increased mucosal eosinophil presence and activation, alongside other immune regulators such as mast cells, are proposed drivers for hyperpermeability [43].

### 4.3. Dysbiosis

Several studies have investigated the role of the gut microbiota in the development of IBS and IBD, and how the microbiota might be modulated for therapeutic effect [52,53]. Indeed, shotgun metagenomic sequencing can differentiate IBS from IBD based on bacterial taxonomy, metabolic functions, antibiotic resistance genes, virulence factors, and bacterial growth rates [54].

Unfortunately, to our knowledge, no studies have yet investigated differences in the gut microbiota between individuals with quiescent IBD who have IBS-type symptoms and those presenting with isolated IBS or IBD. Such studies could elucidate whether IBS-type symptoms in IBD represent a distinct microbial signature and identify novel microbial targets for intervention aimed at alleviating persistent gastrointestinal symptoms in this population.

### 4.4. Gut–Central Nervous System Neural Processing

The enteric nervous system comprises two major plexuses: the myenteric plexus and the submucosal plexus [55]. Intestinal inflammation can lead to the loss of enteric neurons and induce morphological changes in enteric ganglia (that is, clusters of nerve cells located within the wall of the gastrointestinal tract) [56,57], potentially contributing to visceral hypersensitivity and symptoms in IBS [58]. Psychological stress has also been shown to alter enteric neuronal function, glial activity, and gut motility, which may contribute to persistent IBS-type symptoms in patients with quiescent IBD [59]. To date, however, the role of the enteric nervous system among patients with quiescent IBD who have IBS-type symptoms has not been contrasted with IBS or IBD alone.

## 5. General Approaches to Managing IBS in IBD

A relapse of IBD should always be the first consideration when assessing IBD patients with an acute disturbance in bowel function. Once a flare-up of mucosal inflammation is excluded and the need to escalate immunosuppressive therapy is obviated, a detailed clinical history and judicious use of medical tests should be taken to exclude risk factors for alternative acute causes of gastrointestinal symptoms. Among other causes, acute disturbances in gastrointestinal dysfunction may arise from adhesions, renal or gallbladder calculi, or abscesses.

A “positive” approach, that is, one that does not rely on a process of exclusion, should be used to formulate a diagnosis of IBS among patients with chronic gastrointestinal symptoms. A “positive” approach focused on characteristic symptoms may enhance patient acceptability by validating experiences, reducing anxiety, and enabling earlier, targeted management [60,61]. However, adopting a positive diagnostic approach to IBS can be challenging in patients with IBD, as they are at increased risk of chronic gastrointestinal symptoms caused by other conditions that may mimic IBS, such as bile acid malabsorption (particularly in those with ileal disease), SIBO, and celiac disease (Figure 3) [8]. Methods for conveying a “positive” DGBI diagnosis, along with additional strategies for establishing rapport with patients, have been described in detail elsewhere [62,63,64].

**Figure 4:**
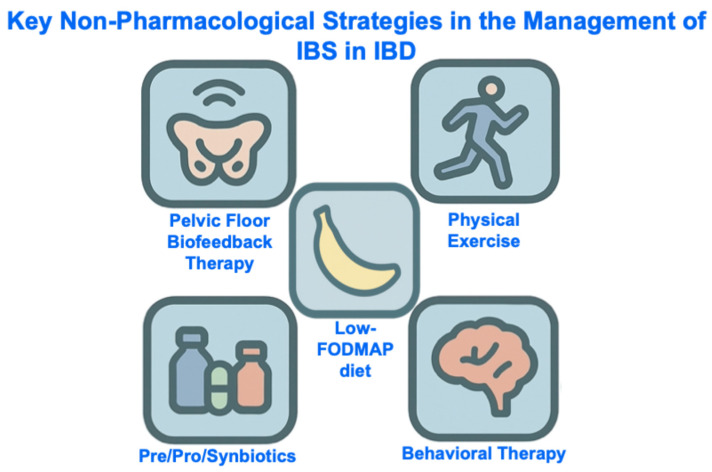
Key Non-Pharmacological Strategies in the Management of IBS in IBD. Abbreviations: IBD, inflammatory bowel disease, IBS, irritable bowel syndrome; FODMAP, Fermentable Oligo-, Di-, Mono-saccharides And Polyols.

**Figure 5:**
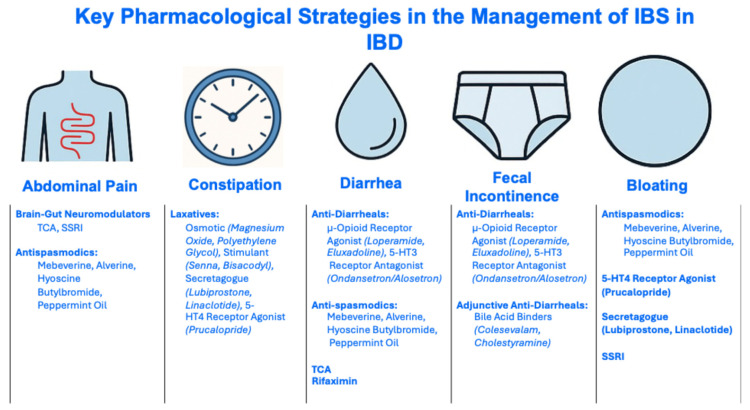
Pharmacological Strategies in the Management of IBS in IBD. Abbreviations: IBD, inflammatory bowel disease, IBS, irritable bowel syndrome; SSRI, Selective Serotonin Reuptake Inhibitor; TCA, tricyclic antidepressant; 5-HT4, 5-Hydroxytryptamine 4.

Gut-directed psychological therapy [65,66], the low-FODMAP (fermentable oligosaccharides, disaccharides, monosaccharides and polyols) diet [67,68,69,70], probiotics [71,72], and ramosetron [73] (Table 1) are the only interventions that have been specifically tested in randomized controlled trials for the management of IBS-type symptoms in patients with quiescent IBD. There is no compelling evidence to suggest that IBS in quiescent IBD should be managed any differently to IBS in the absence of IBD, so general principles for managing IBS, in line with guidance from international bodies, should be followed. This includes a combination of non-pharmacological (e.g., diet, physical activity, psychological therapy) and pharmacological strategies (e.g., neuromodulators, antispasmodics, antidiarrheals, laxatives) which together address the biopsychosocial model [74] (Figure 4 and Figure 5).

**Figure 6:**
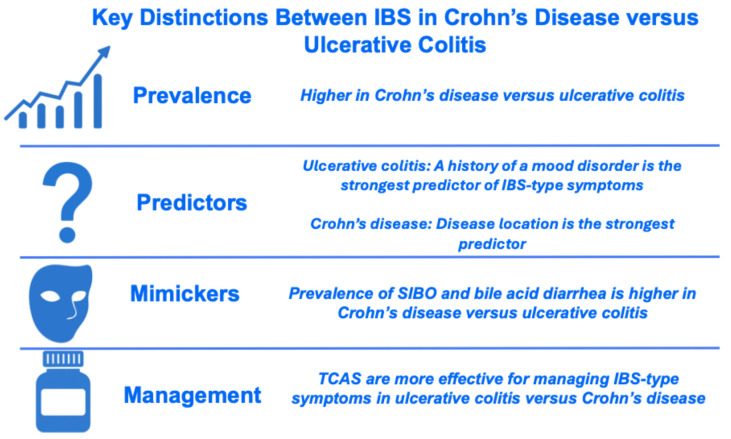
Key Differences Between IBS-type symptoms in Crohn’s Disease versus Ulcerative Colitis. Abbreviations: SIBO, small intestinal bacterial overgrowth; TCA, tricyclic antidepressant.

Particular attention should be paid to psychological factors, since up to one third and one quarter of patients with IBD are affected by anxiety and depression, respectively [75], and both conditions are strongly associated with IBS in IBD [76,77,78]. Multivariable Cox regression demonstrates that a history of mood disorder is the strongest predictor of IBS-type symptoms in ulcerative colitis (HR 5.2 [95% CI 2.2–12.3], *p* = 0.0001), whereas a similarly strong association is not observed in Crohn’s disease [44] (Figure 6).

Brain–gut behavioral therapies are short-term, clinician-delivered, nonpharmacological interventions designed to alleviate gastrointestinal symptoms, with potential additional benefits for coexisting psychological comorbidities [79]. Meta-analyses support the use of brain–gut behavioral therapies for the treatment of global IBS symptoms [80], as well as abdominal pain specifically [81]. While brain–gut behavioral therapies are suggested for patients who are refractory to conventional treatment for 12 months, earlier referral should be made for patients based on accessibility and preference [82]. Behavioral therapies may be delivered in-person or virtually, e.g., mobile phone- or web-based applications [83].

Neuromodulators, particularly low-dose tricyclic antidepressants (TCAs), improve outcomes in IBS [84] and appear effective for IBS-type symptoms in IBD, with patients with ulcerative colitis deriving greater benefit than those with Crohn’s disease [85] (Figure 6). Approaches to using neuromodulators in the management of DGBIs are described by the Rome Foundation [86] and are addressed below (Figure 5 and Section 6).

## 6. Management of Key Gastrointestinal Symptoms

### 6.1. Abdominal Pain

At least one in four patients with quiescent IBD report chronic abdominal pain [76,87], and 42% of patients with IBD would “definitely” like help for pain [88]. Abdominal pain is probably more common among patients with quiescent Crohn’s disease (31.2% mild pain, 15.5% moderate/severe pain) than in those with quiescent ulcerative colitis (23.4% mild pain, 10.1% moderate/severe pain) [89]. The presence of abdominal pain in quiescent IBD is associated with impaired quality of life [90]. Unfortunately, there is “very low” certainty of evidence supporting the use of non-pharmacological and pharmacological therapies in the treatment of abdominal pain in Crohn’s disease and ulcerative colitis, as demonstrated in Cochrane reviews [91,92].

#### 6.1.1. Non-Pharmacological Management of Abdominal Pain

##### Brain–Gut Behavioral Therapy

Patients with chronic abdominal pain in IBD are more likely to report anxiety and depression compared with individuals without abdominal pain [76,87], with at least six studies examining the effectiveness of behavioral therapies [93]. Among these six studies, relaxation techniques and changing cognitions are arguably the most promising behavioral interventions [93]. Recently, the IBD boost study (a UK-based, pragmatic two-arm, parallel group randomized controlled trial of a 12-session facilitator-supported online cognitive behavioral self-management program, versus care as usual, to manage symptoms of fatigue, pain and fecal urgency/incontinence in IBD [94]) demonstrated no change in abdominal pain scores at six months in experimental group participants versus the care as usual arm [95].

##### Low-FODMAP Diet

Among dietary approaches for IBS, the low-FODMAP diet has the most robust evidence base [96]. The low-FODMAP diet is a restrictive diet that first excludes all food products containing FODMAPs and later re-introduces the FODMAPs one by one. The efficacy of the low-FODMAP diet in reducing abdominal pain among patients with IBD is supported by meta-analyses [97,98,99], and has been confirmed in two prospective studies of patients specifically with quiescent IBD [67,100].

The low-FODMAP diet, if recommended, should be followed under the supervision of a registered dietitian, given the high prevalence (approximately 20%) of patients with IBD who have a positive screening for avoidant–restrictive food intake disorder [101]. Recently, a multicenter randomized noninferiority clinical trial has shown that the Mediterranean diet is a viable first-line dietary intervention for IBS, but this has yet to be confirmed among patients with IBS and quiescent IBD [102].

##### Physical Exercise

Evidence supports the role of physical activity in managing symptoms of IBS [103], but whether exercise can help manage co-morbid IBS in patients with IBD is unproven [104].

#### 6.1.2. Pharmacological Management of Abdominal Pain

##### Opioids

The use of opioids in the management of chronic abdominal pain is discouraged [105] and there is no evidence that opioids improve abdominal pain or quality of life in patients with Crohn’s disease [106]. More broadly, opioid use among patients with IBS is associated with greater psychological impairment and healthcare utilization [107], and may trigger or exacerbate other disorders of gut–brain interaction, such as cyclical vomiting syndrome [108], functional dyspepsia [109], and opioid-induced gastrointestinal hyperalgesia (e.g., narcotic bowel syndrome) [110]. For these reasons, opioid use should be discouraged in the management of chronic abdominal pain in IBD.

##### Brain–Gut Neuromodulators

A meta-analysis of 28 randomized controlled trials supports the use of TCAs for ongoing global symptoms or abdominal pain in IBS, but also highlights a potential for selective serotonin reuptake inhibitors (SSRIs) to be modestly effective for abdominal pain [111]. A moderate improvement in global well-being scores has been observed among patients with quiescent IBD with IBS, over 80% of whom report abdominal pain, who were treated with TCAs [85]. TCAs have an affinity to multiple different receptors which may result in symptoms including but not limited to dry mouth, drowsiness, blurred vision, fatigue, and constipation (Section 6.2) [112].

##### Antispasmodics

Antispasmodics, including hyoscine, dicyclomine, and peppermint oil, are thought to relieve symptoms of IBS by reducing smooth muscle contraction and possibly visceral hypersensitivity [113]. There is “very low” quality evidence supporting the use of antispasmodics in the treatment of abdominal pain in IBS [114], and indirect comparisons suggest that these agents are less effective than TCAs [115].

### 6.2. Constipation

Approximately 50% of patients with ulcerative colitis have an “ulcerative colitis-associated constipation syndrome”, occasionally referred to as “proximal constipation”, which typically occurs in the setting of active left-sided and distal disease [116]. Ulcerative colitis-associated constipation syndrome can be characterized by a reduced frequency of defecation, passage of hard or dry stools, straining, and/or the sensation of incomplete evacuation [117]. Wherever feasible, symptoms of constipation should be assessed using prospectively completed bowel diaries, not questionnaires completed at a single time point [118]. Possible causes of constipation in IBD may relate to derangement of colonic innervation, impairment of mucous production, or anorectal dyssynergia [119].

#### 6.2.1. Non-Pharmacological Management of Constipation

##### Diet

Guidelines do not recommend any whole diet approaches (e.g., high-fiber diet) for the treatment of constipation due to lack of evidence [120]. Most recommendations for diet adjustments for constipation are supported by evidence of “very low” or “low” quality [120]. In a retrospective study, the low-FODMAP diet did not improve constipation in patients with IBD [121].

##### Physical Exercise

Insufficient physical activity is associated with an increased risk of constipation [122], which suggests that exercise may be an effective strategy for the treatment of constipation. Fatigue features prominently in both IBD and IBS [123] and physical activity may help reduce fatigue in IBD [124,125].

##### Pelvic Floor Biofeedback Therapy

Defecatory disorders are defined as difficulty in evacuating stool from the rectum in patients with chronic or recurring symptoms of constipation [126,127,128]. These disorders arise secondary to a combination of abnormal anal sphincter pressures, impaired anal relaxation, and inadequate rectal propulsive forces [129]. In patients with ongoing defecatory symptoms, the prevalence of a functional defecatory disorder (after excluding cases of obstructive defecation due to stricture or malignancy) among patients with IBD without an ileal pouch–anal anastomosis (IPAA) ranges from 45% to 97%, and in patients with IPAA, it can range from 25% to 75% [130]. This subset of patients should undergo further evaluation with defecography to exclude structural causes of outlet obstruction, such as internal intussusception, solitary rectal ulcer syndrome, rectocele, rectal prolapse, or, in IPAA patients, floppy pouch complex.

Biofeedback therapy, a learning process centered on operant conditioning, is a key treatment option for individuals with defecation disorders. Biofeedback therapy aims to (i) educate patients about disordered defecation; (ii) coordinate increased intra-abdominal pressure with pelvic floor muscle relaxation during evacuation; and (iii) practice simulated defecation with a balloon, aided by a therapist [131]. The response rate to biofeedback therapy in patients with IBD with and without IPAA is 86% and 70%, respectively [130].

#### 6.2.2. Pharmacological Management of Constipation

Laxatives can be used to treat constipation, and guidelines generally suggest that these should be introduced in a stepwise fashion [132,133]. Classes of laxatives include osmotic laxatives (e.g., magnesium oxide or polyethylene glycol), stimulant laxatives (e.g., senna or bisacodyl), secretagogues (e.g., lubiprostone [unavailable in Europe] or linaclotide), and selective serotonin 5-HT4 agonists (e.g., prucalopride). The safety profiles of laxatives are described elsewhere [134].

### 6.3. Diarrhea

A greater proportion of patients with IBS-type symptoms in quiescent IBD fulfill the criteria for IBS-D than IBS-C (75.4% vs. 14.0%) [44]. Diarrhea, not necessarily in the context of IBS, has been studied among patients with quiescent IBD in the UK [135] and USA [136]. In a UK sample, a higher proportion of patients with quiescent IBD report diarrhea “all/most of the time” (15% vs. 12.1%) and “sometimes” (48.5% vs. 34.8%) than constipation. In a USA sample, 30.6% of patients report greater than 4 bowel movements daily [136].

The pathogenesis of diarrhea in patients with quiescent IBD is associated with reduced microbial diversity [137] and potentially increased colonic propulsive activity [138], although this is unproven in patients with inactive mucosal inflammation.

#### 6.3.1. Non-Pharmacological Management of Diarrhea

For the treatment of IBS-D and functional diarrhea, European investigators achieved a strong consensus for the use of a low-FODMAP diet and gut-directed psychological therapies [139]. Evidence also supports the use of physical activity and prebiotics, probiotics, or synbiotics [140].

#### 6.3.2. Pharmacological Management of Diarrhea

The American Gastroenterology Association has provided conditional recommendations for eluxadoline [not available in Europe], rifaximin, alosetron, (moderate certainty) [not available in Europe], loperamide (very low certainty), TCAs, and antispasmodics (low certainty) [141].

Loperamide and eluxadoline (strictly not to be taken by patients without a gallbladder or in patients with known or suspected biliary tree or pancreatic duct obstruction) are opioid receptor agonists which inhibit intestinal motility.

The efficacy of rifaximin, a non-absorbable antibiotic which is FDA-approved for the treatment of IBS-D, has been confirmed in a meta-analysis of five controlled trials (NNT of 9) [142], and its use for this indication is endorsed by North American guidelines [143].

Alosetron is a selective 5-HT3 antagonist which is FDA-approved for the treatment of IBS-D among females, although there are no sex-specific reasons justifying its use among women [144]. There is also convincing evidence supporting the use of ondansetron, an alternative 5-HT3 antagonist, in the treatment of IBS-D [145,146].

A post-hoc analysis of the pivotal ATLANTIS trial demonstrated that patients with IBS-D were more likely to respond to low-dose TCA than those without this subtype [147], supporting the use of this agent among those with loose stools. TCAs may exert some of their therapeutic effects in patients with diarrhea through their antihistaminic properties [86]. This notion is further supported by evidence that ebastine, a selective H1-receptor antagonist, reduces the frequency of loose stools in individuals with IBS [148]. Collectively, these observations highlight the need to examine the contributions of histamine production and mast-cell activity to the pathogenesis of persistent diarrhea in inactive IBD.

Much of the evidence supporting the use of antispasmodics in the treatment of IBS-D is supported by “global relief of IBS symptoms”. However, in the secondary analyses of a four-center study comparing the use of small-intestinal-release peppermint oil, ileocolonic-release peppermint oil, or placebo in the treatment of IBS, no significant changes were observed in stool consistency and frequency between the three arms [149], suggesting no improvement in diarrhea. In patients with IBD, antispasmodic use has been linked to higher rates of opioid use and persistent abdominal pain, even after adjusting for disease severity [150]. This raises concerns that these agents may be ineffective and, in some cases, could exacerbate symptoms, highlighting the need for caution when considering their use.

### 6.4. Fecal Incontinence

Fecal incontinence (FI), defined as the recurrent uncontrolled passage of fecal material [151], affects at least 14% of patients with IBD [152], a prevalence nearly twice that observed in community-dwelling adults [153]. Altered bowel movements, particularly diarrhea, are strongly associated with FI [154], and this phenomenon is observed among patients with IBD and FI [152]. Features of FI—such as sensation preceding leakage and the volume and consistency of the leakage—are best assessed using a prospective diary rather than a single time-point questionnaire [155]. Unsupervised cluster analysis has identified four phenotypes of stool consistency and bowel patterns among patients with FI [156] and similar analyses should be replicated in individuals with FI in the context of IBD and IBS to help tailor treatment.

Causes of FI in patients with IBD may be secondary to reduced rectal compliance (that is, a rectum that cannot retain a sufficient volume stool) [157], perianal Crohn’s disease, and/or external anal sphincter weakness [158].

#### 6.4.1. Non-Pharmacological Management of Fecal Incontinence

Non-surgical, non-pharmacological treatment strategies for FI include basic behavioral advice (toilet routine and bowel training), lifestyle adjustments (maintaining a healthy body mass index), pelvic floor biofeedback therapy, transanal irrigation and barrier inserts [159]. Surgical options can include sacral neuromodulation and anal dextranomer injection, with colostomy or anal sphincteroplasty rarely required now [160].

#### 6.4.2. Pharmacological Management of Fecal Incontinence

Since diarrhea and rectal urgency are key risk factors of FI [161], antidiarrheal agents (e.g., bile acid binders, TCAs, the 5-HT3 receptor antagonist ondansetron, and the opioid receptor agonists eluxadoline and loperamide) are important pharmacological therapies to manage FI in patients [162].

### 6.5. Abdominal Bloating

Abdominal bloating is the subjective sensation of fullness, swelling, or trapped gas perceived in any region of the abdomen, in contrast to abdominal distention, which refers to a visible increase in abdominal girth often described as “like a balloon” or “like being pregnant” [163]. At least one in five patients with quiescent IBD report bloating [85,135] at a rate not significantly greater than in patients with IBS [85].

#### 6.5.1. Non-Pharmacological Management of Abdominal Bloating

##### Diet

The low-FODMAP diet has not been explicitly assessed for the treatment of functional bloating and distention [163]; however, trials in functional dyspepsia and IBS comparing it with traditional dietary advice have shown benefits in bloating and quality of life [164,165,166]. Bloating is considered the most responsive symptom to the low-FODMAP diet in patients with IBD and DGBI symptoms [99].

##### Brain–Gut Behavioral Therapy

While much of the evidence supporting brain–gut behavioral therapies is in the context of IBS, psychological therapies may be beneficial across the spectrum of DGBIs, including those characterized by bloating [79]. In one randomized controlled trial, an eight-week course of mindfulness-based stress reduction resulted in an improvement in bloating severity at three months post-course completion [167].

##### Biofeedback Therapy

A proportion of patients with bloating and/or visible abdominal distention have abdominophrenic dyssynergia; a maladaptive neuromuscular response characterized by diaphragmatic contraction and abdominal wall relaxation [168]. Abdominothoracic wall movements serve as an effective biofeedback signal for correcting abdominophrenic dyssynergia and abdominal distention [169]. One study suggests that over 60% of patients who experience bloating and are diet non-responders have features of a defecatory disorder, which may respond favorably to pelvic floor biofeedback therapy [170].

#### 6.5.2. Pharmacological Management of Abdominal Bloating

While rifaximin can reduce symptoms of abdominal bloating [171], a two-week course of this non-absorbable antibiotic is only FDA-approved for the treatment of IBS-D. Meta-analyses support the use of antispasmodics [172,173] and prokinetics [174] in the treatment of bloating among select patients with constipation. Secretagogues [175,176,177] and SSRIs [178] may also be helpful for bloating.

## 7. Future Directions

### 7.1. Advancing Diagnostic Precision

Current clinical tools may not reliably distinguish a “true” DGBI from subtle inflammatory activity. Therefore, future studies should evaluate multimodal biomarkers, including mucosal immunophenotyping, microbiota and metabolomic signatures, neuroimaging, and measures of barrier function, to develop biomarker-based diagnostic approaches rather than relying solely on symptoms.

### 7.2. Mechanistic and Longitudinal Research

Longitudinal cohort studies integrating biological, behavioral, and environmental data are needed to map trajectories of symptom development, identify prognostic markers, and determine whether early control of inflammation, stress, and diet might be able to modify risk.

### 7.3. Targeted Therapeutic Trials

Randomized trials specifically designed for patients with IBD who have IBS-type symptoms are required. These should assess diet-based interventions (e.g., low-FODMAP, Mediterranean diets), gut-directed psychological therapies, approaches to neuromodulation (including dose-guided TCA strategies), and biofeedback for pelvic floor disorders and/or abdominophrenic dyssynergia. Treatment studies should incorporate mechanistic endpoints to support precision care, including microbiota shifts, central pain processing biomarkers, and advanced MRI measures. MRI can be used to non-invasively quantify small bowel water content [179], intestinal motility [180], and intestinal permeability [181]. Integrating these imaging endpoints allows researchers to link physiological changes with symptom improvement, providing mechanistic insight into how interventions like TCAs or diet can modify gut function.

### 7.4. Holistic Outcome Assessment

Therapeutic trials should move beyond gastrointestinal symptoms alone to include measures of fatigue, sleep, mood, cognitive symptoms, and functional impairment, reflecting the multidimensional burden experienced by this population.

### 7.5. The Patient-Physician Relationship

Accurately identifying IBS-type symptoms in patients with IBD requires a positive diagnostic approach rather than relying on a diagnosis of exclusion. Training physicians to adopt this approach should begin at the medical school level [182,183], ensuring that future clinicians learn to recognize IBS as a distinct clinical entity even in the context of IBD. Incorporating simulated patient encounters, in which learners engage with adaptable, realistic patient scenarios, can reinforce both diagnostic skills and patient-centered communication [184].

## 8. Conclusions

IBS-type symptoms affect approximately one in three patients with quiescent IBD, significantly impairing quality of life and posing diagnostic challenges for clinicians, who must differentiate these symptoms from conditions such as SIBO, bile acid malabsorption, celiac disease or other post-inflammatory complications. The coexistence of IBS and IBD can be viewed as a complex “matrimony” between functional and organic gastrointestinal disorders, arising from a dynamic interplay of residual inflammation, altered brain–gut signaling, dysbiosis, and psychosocial impairment. A positive, symptom-focused diagnostic approach, combined with careful exclusion of active inflammation, should be used to guide management. Current evidence supports the use of dietary interventions, brain–gut behavioral therapies, and neuromodulators, applied according to general IBS management principles, while also addressing the psychosocial burden of disease. Future studies should use more precise biomarkers to detect ongoing inflammation and track patients over time to improve diagnosis and guide better care for this group.

## Figures and Tables

**Table 1:** Studies evaluating interventions for IBS-type symptoms in IBD. Abbreviations: CAI, clinical activity index; CD, Crohn’s disease; CDAI, clinical disease activity index; CRP, c-reactive protein; DGBI, disorder of gut–brain interaction; FODMAP, Fermentable Oligo-, Di-, Mono-saccharides and Polyols; HBI, Harvey–Bradshaw Index; IBD, inflammatory bowel disease; IBDQ, inflammatory bowel disease questionnaire; IBS, irritable bowel syndrome; IBS-SSS, irritable bowel syndrome severity scoring system; MCT, multi-convergent therapy; QoL, quality of life; RCT, randomized controlled trial; SCCAI, simple clinical colitis activity index; SIBDQ, short inflammatory bowel disease questionnaire; UC, ulcerative colitis.

First Author, Year, Reference	Study Design, IBD Population (Crohn’s Disease [CD] vs. Ulcerative Colitis [UC]), Number of Patients	Intervention Studied	Definition of Inflammatory Bowel Disease (IBD) Remission	Definition of Irritable Bowel Syndrome (IBS)	Any Other Disorder of Gut–Brain Interaction (DGBI) Considered in the Study?	Main Findings
Berrill et al., 2014 [65]	Non-blinded RCT of 66 IBD patients	MCT (multi-convergent therapy)—a form of psychotherapy	SCCAI < 3 in UC (modified to exclude ‘general wellbeing’); HBI < 5 in CD; CRP < 10 mg/L	Rome III	No	In IBD patients with IBS-type symptoms, there was a significantly higher mean IBDQ score in the MCT group compared to controls. Stepwise linear regression analysis of the MCT group found the only significant factor associated with an improved inflammatory bowel disease questionnaire (IBDQ) score at four months to be the presence of IBS-type symptoms. A high drop-out rate was noted in the MCT group, with 24% failing to attend a single appointment, suggesting this intervention may not be acceptable to all.
Pedersen et al., 2017 [67]	Open-label randomized controlled trial (RCT) of 89 patients with IBD	Low-FODMAP diet	Absence of recent relapse, steroid use or therapy adjustment; simple clinical colitis activity index (SCCAI) ≤ 2 and fecal calprotectin < 100 μg/g in UC; Harvey–Bradshaw Index (HBI) < 5 and fecal calprotectin < 200 μg/g in CD; c-reactive protein (CRP) ≤ 10 mg/L	Rome III	No	After six weeks of the low-FODMAP diet, significantly lower median irritable bowel syndrome severity scoring system (IBS-SSS) and greater SIBDQ scores were demonstrated when compared to habitual diet. Subgroup analysis revealed improvement in IBS-SSS was limited to patients in inflammatory remission and statistical significance was noted only in CD, not UC. UC treated with a low-FODMAP diet did, however, show improvement in disease activity (evaluated using the SCCAI).
Cox et al., 2017 [68]	Double-blind, placebo-controlled, cross-over RCT of 32 IBD patients	Low-FODMAP diet	Fecal calprotectin < 250 μg/g; CRP < 10 mg/L	Rome III	Functional bloating and functional diarrhea (Rome III)	Participants with a previous response to low-FODMAP diet and DGBI were randomized and re-challenged with a series of three-day FODMAP trials. The incidence and severity of abdominal pain, bloating and flatulence were all significantly higher across FODMAP challenges, as was severity of fecal urgency. Post-hoc analysis revealed that this effect was limited to fructans, rather than galacto-oligosaccharides or sorbitol.
Bodini et al., 2019 [69]	RCT of 55 IBD patients	Low-FODMAP diet	Mayo score < 6 in UC; HBI < 8 in CD	Rome IV	No	After the six-week dietary intervention, median HBI, median fecal calprotectin and Mayo scores were decreased in the low-FODMAP diet group versus baseline. This was not observed in the standard diet group.
Cox et al., 2020 [70]	Single-blind RCT of 52 IBD patients	Low-FODMAP diet	Physician global assessment; stable medications with no recent IBD flares; Fecal calprotectin < 250 μg/g; CRP < 10 mg/L	Rome III	Functional bloating and functional diarrhea (Rome III)	A higher proportion of patients reported adequate relief of gut symptoms following four weeks of low-FODMAP diet versus sham diet although total IBS-SSS score was not significantly different across both groups. Subgroup analysis of UC revealed a significantly greater reduction in IBS-SSS score with the low-FODMAP diet compared to sham diet. This was not reproduced in CD.
Hoekman et al., 2020 [66]	Open-label RCT of 80 IBD patients	Hypnotherapy	Physician’s global assessment and fecal calprotectin ≤ 100 µg/g, or ≤200 µg/g without inflammation at endoscopy	Rome III	No	There was no statistically significant difference in the reduction in IBS-SSS, QoL, depression and anxiety scores at week 40 between patients who received hypnotherapy and those who received standard medical therapy.
Lee et al., 2022 [71]	Observational study of 43 patients with UC	Biotop capsule^®^ (*Lactobacillus acidophilus*, *Clostridium butyricum* TO-A, *Bacillus mesentericus* TO-A and *Streptococcus faecalis* T-110)	Mayo endoscopic score ≤ 1	Rome IV	No	Four weeks of treatment with the probiotic Biotop capsule^®^ was delivered. Small, but statistically significant, improvements in self-reported bowel function, systemic function and social function were observed (assessed using the short inflammatory bowel disease questionnaire [SIBDQ]). There was no change in emotional function. A small improvement in stool consistency, measured using the Bristol stool scale, was also noted.
Tomita et al., 2022 [73]	Double-blind, placebo-controlled RCT of 70 IBD patients	Ramosetron	CAI ≤ 4 in UC; CDAI ≤ 150 in CD CRP ≤ 0.3 mg/dL	Rome III—Japanese version	No	The responder rate for relief from overall IBS-D-like symptoms and for improvement in bowel habit was significantly higher in the ramosetron group than in the placebo group. Reduction in stool frequency was significantly greater in the ramosetron group than in the placebo group.
Tomita et al., 2023 [72]	Prospective pilot study of 12 patients with CD	*Bifidobacterium bifidum* G9-1 (BBG9-1)	Endoscopy; CD activity index (CDAI) ≤ 150; CRP ≤ 0.3 mg/dL	Rome III—Japanese version	No	Four weeks of treatment with probiotic BBG9-1 yielded an improvement in the CDAI score, IBS severity index, and self-reported gastrointestinal symptoms. None of these results were statistically significant. There was a significant improvement in IBD-related QoL and anxiety scores, suggesting a favorable impact on mental health. Fecal calprotectin remained unchanged implying that improvements were not related to improved inflammatory burden.

## Data Availability

No new data were created or analyzed in this study.

## Abbreviations

CAI	Clinical activity index
CD	Crohn’s disease
CDAI	Clinical disease activity index
CRP	C-reactive protein
DGBI	Disorder of Gut–Brain Interaction
FODMAP	Fermentable oligosaccharides, disaccharides, monosaccharides and polyols
FDA	Food and Drug Administration
FI	Fecal incontinence
HBI	Harvey–Bradshaw Index
IBD	Inflammatory bowel disease
IBDQ	Inflammatory bowel disease questionnaire
IBS	Irritable bowel syndrome
IBS-C	Irritable bowel syndrome—constipation predominant
IBS-D	Irritable bowel syndrome—diarrhea predominant
IBS-SSS	Irritable bowel syndrome—severity scoring system
IPAA	Ileal pouch–anal anastomosis
MCT	Multi-convergent therapy
PROM	Patient-reported outcome measure
QoL	Quality of life
RCT	Randomized controlled trial
SCCAI	Simple clinical colitis activity index
SIBDQ	Short inflammatory bowel disease questionnaire
SIBO	Small intestinal bacterial overgrowth
SSRI	Selective serotonin reuptake inhibitor
TCA	Tricyclic antidepressant
UC	Ulcerative colitis
